# 3D Printing of Ultrathin MXene toward Tough and Thermally Resistant Nanocomposites

**DOI:** 10.3390/nano12162862

**Published:** 2022-08-19

**Authors:** Yuewei Li, Ranjith Kumar Kankala, Ai-Zheng Chen, Shi-Bin Wang

**Affiliations:** 1 Institute of Biomaterials and Tissue Engineering, Huaqiao University, Xiamen 361021, China; 2 Fujian Provincial Key Laboratory of Biochemical Technology, Huaqiao University, Xiamen 361021, China

**Keywords:** 3D printing, photosensitive resin, mechanical property, thermal resistance

## Abstract

Liquid crystal display (LCD)-based 3D printing, a facile and cost-effective manufacturing technique, is often applied when fabricating objects with porcelain structures using photosensitive resins (PSRs). Currently, 3D printed constructions are typically used as models for demonstration purposes rather than industrial applications because of their poor performance. In this study, we prepared nanocomposites by incorporating Ti_3_C_2_ MXene nanosheets to enhance the overall characteristics of a PSR, including mechanical properties and thermal resistance. Notably, the designed nanocomposites showed optimum performance at an MXene loading of 0.5% *w*/*w*. The mechanical properties of the designed nanocomposites confirmed the enhanced ultimate tensile and flexural strengths (by 32.1% and 42.7%, respectively), at 0.5% *w*/*w* MXene loading. Moreover, the incorporated MXene presented no substantial influence on the toughness of the PSR. The glass transition and thermal degradation temperatures at 5% weight loss increased by 7.4 and 10.6 °C, respectively, resulting predominantly from the hydrogen bonding between the PSR and MXene. Together, the experimental results indicate that the designed PSR/MXene nanocomposites are expected to replace pristine resins for LCD printing in various practical applications.

## 1. Introduction

Additive manufacturing (AM), also referred to as 3D printing, is a facile, rapid, and cost-effective method for fabricating 3D objects with porcelain structures in a layer-by-layer manner for personalized customization [1,2]. Owing to its unique advantages, 3D printing has been employed in diverse applications, such as catalysis [3], electronic sensors [4], aerospace [5], and tissue engineering [6,7]. Various 3D printing technologies have been developed to meet different requirements, such as those for fused filament fabrication, digital light processing (DLP), stereolithography (SLA), liquid crystal display (LCD), selective laser sintering, powder bed fusion, and inkjet printing. Among the various 3D printing technologies available, vat polymerization, involving SLA, DLP, and LCD [8], is the most widely applied technique because of the excellent surface finish quality and the precision of the printed workpieces produced at high printing speed [9]. Specifically, the use of LCD technology in imaging systems has garnered significant interest from researchers for manufacturing inexpensive LCD printers [10].

Currently, 3D printed parts, which are predominantly used as prototypes, suffer from poor performance, hindering their practical translation to the industrial scale. Therefore, obtaining photosensitive resins (PSRs) with good overall properties for LCD 3D printing is crucial. As the PSRs undergo selective UV-curable reactions during the printing process [11], the strategy for enhancing the properties can be derived from the UV-curable resins of other vat polymerization technologies. To enhance the properties of the UV-curable resins, most efforts have focused on the molecular design of polymer chains [12] and the introduction of nanofillers [13]. Fabricating effective nanocomposites involves combining various materials to obtain the desired properties, thereby overcoming the performance limitations of the polymers in pristine materials. Several nanofillers that enhance the mechanical properties and thermal resistance of 3D-printed workpieces from vat polymerization have been investigated, including graphene [14], nanofibers [15], and inorganic oxide nanoparticles [16].

Among the nanomaterials, transition metal carbides/nitrides (MXene) have recently emerged as novel two-dimensional nanomaterials, which are of specific interest in engineering-based applications [17]. MXene nanosheets have been verified to be promising nanofillers for improving the thermal and mechanical properties of polymers. In a study by Shi et al. [18], the prepared MXene/polypropylene nanocomposites enhanced the initial degradation temperature (by 79.1 °C) and tensile strength (by 35.3%) compared with the pristine material. Similarly, by introducing MXenes, the tensile strength of thermoplastic polyurethane nanocomposites improved by 41.2% at 0.5 wt% filler loading, and the transfer of gas molecules from the nanocomposites was hindered at high temperatures [19]. For 3D printing, MXene has been used in supercapacitor preparation [20] and bone reconstruction [21,22]. However, the effects of MXene on UV-curable systems for 3D printing are of less concern.

In this study, MXene-based PSR nanocomposites were prepared to explore the effect of the MXene on LCD 3D printing. Initially, MXene was etched and exfoliated from Ti_3_AlC_2_ and then characterized by transmission electron microscopy (TEM), atomic force microscopy (AFM), and X-ray diffraction (XRD). Then, the PSR/MXene nanocomposites were produced using a commercial LCD printer. Furthermore, the effects of MXene on the mechanical and thermal performances of the designed nanocomposites were systematically explored. The MXene-reinforced nanocomposites prepared in this work could pave the way for novel PSRs and broaden the use of LCD 3D printing in extended industrial applications.

## 2. Materials and Methods

### 2.1. Materials

Urethane diacrylate oligomer (CN1964 NS) and (4) ethylene oxide pentaerythritol tetraacrylate (SR494 NS) were provided by Sartomer Co., Ltd. (Guangzhou, China). Diphenyl (2,4,6-trimethyl benzoyl) phosphine oxide (TPO), poly(ethylene glycol) diacrylate (PEGDA-200, M_w_ ~200), and lithium fluoride (LiF, AR 99%) were obtained from Aladdin Reagent Co., Ltd. (Shanghai, China). Ti_3_AlC_2_ (400 mesh) was supplied by XinXi Technology Co., Ltd. (Foshan, China), and concentrated hydrochloric acid (HCl, 35–37% aq.) was obtained from Sinopharm Chemical Reagent Co., Ltd. (Shanghai, China).

### 2.2. Preparation of the Exfoliated MXene Nanosheets

In a Teflon vial, 0.5 g of LiF and 10 mL of 9 M HCl were mixed at room temperature for 20 min. Then, 0.5 g of Ti_3_AlC_2_ raw powder was gradually added to the vial under stirring, and the mixture was heated to 35 °C for 35 h under magnetic agitation to etch the Al interlayers. Further, the residual powders were washed with ultrapure water until the pH of the supernatant exceeded 6. After that, the mixture was subjected to ultrasonication for 45 min to obtain the exfoliated MXene nanosheets. The suspension was centrifuged at 4000 rpm for 10 min to separate the non-exfoliated Ti_3_C_2_, and the colloidal suspension with the exfoliated MXene nanosheets was freeze-dried to obtain a few-layered MXene.

### 2.3. Preparation of the PSR/MXene Nanocomposites

In this study, the pure PSR comprised a mixture of 40 phr of CN1964 NS, 10 phr of SR494 NS, 48 phr of PEGDA-200, and 2 phr of TPO. A specific loading of exfoliated dry MXene nanosheets was dispersed in the diluents (PEGDA-200) via sonication in an ice bath for 30 min. The remaining components of the PSR were then added, and the mixture was stirred at 50 °C for 1 h in the dark. All the specimens were prepared using an LCD printing apparatus (S100, Doumi, Xiamen, China). The thickness of each obtained layer was around 0.05 mm. The irradiation time intervals in the printing process for the base six layers and remaining layers for all the samples were 45 and 10 s, respectively. After 3D printing, the samples were washed several times with ethanol until there was no residual liquid resin on their surfaces.

### 2.4. Characterization

The TEM images were collected using an FEI transmission electron microscope (Hitachi, Japan) operated with an acceleration voltage of 200 kV. In contrast, the AFM images of the exfoliated MXene nanosheets were obtained using a Dimension FastScan atomic force microscope (Bruker Dimension Icon, Hamburg, Germany). The XRD patterns of the samples were obtained through an X-ray diffractometer (Bruker D8 Advance, Germany) with assisted Cu-Kα radiation. The rheological studies were carried out using a rheometer (Discovery Hybrid Rheometer-2, TA, New Castle, DE, USA) with a 40 mm parallel-plate champ for a shear rate ranging from 0.1 to 100 s^−1^ at 25 °C, and the gap between the clamps was 0.5 mm. The mechanical properties, including tensile, flexural, and fracture behaviors of the pure PSR and PSR/MXene nanocomposites, were investigated using a universal material testing machine (AGX-100 plus, Shimadzu, Kyoto, Japan) following the ISO 527, ISO 604, and ASTM 5045 standards. The test speed in tension and flexure was 2 mm/min, and fracture tests were conducted at 10 mm/min. The Izod impact strengths of the pure PSR and PSR/MXene were measured by a Charpy tester (CEAST 9050, Torino, Italy) according to ISO 180. At least five specimens were tested to obtain the average value in mechanical tests, and the tests were performed under conditions of 25 ± 2 °C and 50 ± 5% relative humidity. The nanoindentation analysis was conducted using a nanoindenter (Hysitron Inc., Tribo Indenter 750, Minneapolis, MN, USA) by exploring a matrix of 6 × 6 indentations with a 50 µm distance in each sample. The dispersion of MXene in the PSR matrix was investigated using TEM for observing the ultrathin nanocomposites from an ultrathin microtome machine (EM UC7, Leica, Bensheim, Germany). The microstructure of Ti_3_AlC_2_ before and after etching and the fracture surfaces of the samples after the tensile tests was observed by scanning electron microscopy (SEM, SU 5000, Hitachi, Tokyo, Japan). The samples were covered with a gold layer before observation to improve conductivity. Dynamic mechanical analysis (DMA) of the pristine PSR and PSR/MXene nanocomposites was carried out using a dynamic mechanical analyzer (242E, Netzsch, Bavaria, Germany) in a single cantilever pattern at temperatures ranging from 30 to 180 °C at a heating rate of 3 °C·min^−1^. The test frequency was set to 1 Hz, and the test amplitude was 10 μm. Each specimen was of 35 × 13 × 3 mm in dimensions, and at least three specimens were tested for each material. Thermogravimetric analysis (TGA) was performed using STA449F3 (Netzsch, Bavaria, Germany) from 30 to 800 °C at a 10 °C·min^−1^ heating rate in an air atmosphere.

## 3. Results

### 3.1. Morphology of the MXene Nanosheets

**Figure 1:**
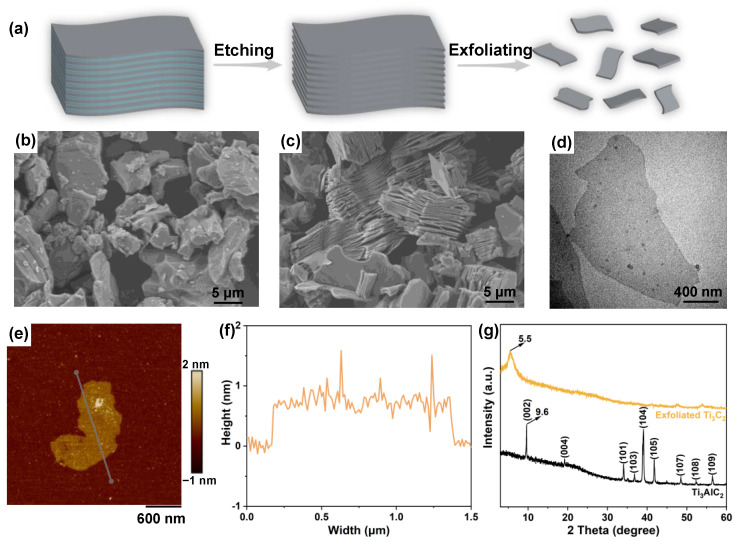
(**a**) Schematic of the synthesis of the MXene nanosheets. SEM images of the Ti_3_AlC_2_ powders (**b**) before and (**c**) after etching. (**d**) TEM and (**e**) AFM images of the MXene nanosheets. (**f**) The corresponding height curve of the exfoliated MXene nanosheets. (**g**) XRD patterns of Ti_3_AlC_2_ and the exfoliated Ti_3_C_2_ nanosheets.

A schematic of the as-prepared exfoliated MXene nanosheets is shown in Figure 1a, which depicts a two-step process. To study the etching effect, the morphologies of Ti_3_AlC_2_ and Ti_3_C_2_ were investigated by SEM. Bulk Ti_3_AlC_2_ raw powders with metallic and covalent/ionic bonds [23] showed a compact layered structure (Figure 1b). After etching with LiF and HCl, the microstructure of Ti_3_C_2_ showed a loosely stacked multilayer structure and a wrinkle-like morphology (Figure 1c). The resultant gaps could be attributed to removing the Al atoms between the interlayers. The morphological features of a single-layer MXene nanosheet were observed using TEM, which indicated excellent transparency (Figure 1d). The high-resolution TEM images (Figure S1) showed that the exfoliated MXene nanosheets formed of a single- or few-layered feature. From the AFM results, the thickness of the exfoliated MXene nanosheets was approximately 2 nm (Figure 1e,f), indicating that the few-layered MXene nanosheets were obtained after the ultrasonication-assisted exfoliation. In the XRD patterns curves (Figure 1g), the peak at the 2θ angle of 39° could be attributed to the (104) lattice plane of Ti_3_AlC_2_, nearly disappearing in the curve of exfoliated Ti_3_C_2_ and stemming from the vanished Al layers [24]. Furthermore, it was observed that the (002) peak moved to a lower 2θ angle, demonstrating a more considerable interlayer distance in the exfoliated Ti_3_C_2_ and providing substantial evidence of the successful formation of the MXene nanosheets.

### 3.2. 3D Printing and Rheological Studies

**Figure 2:**
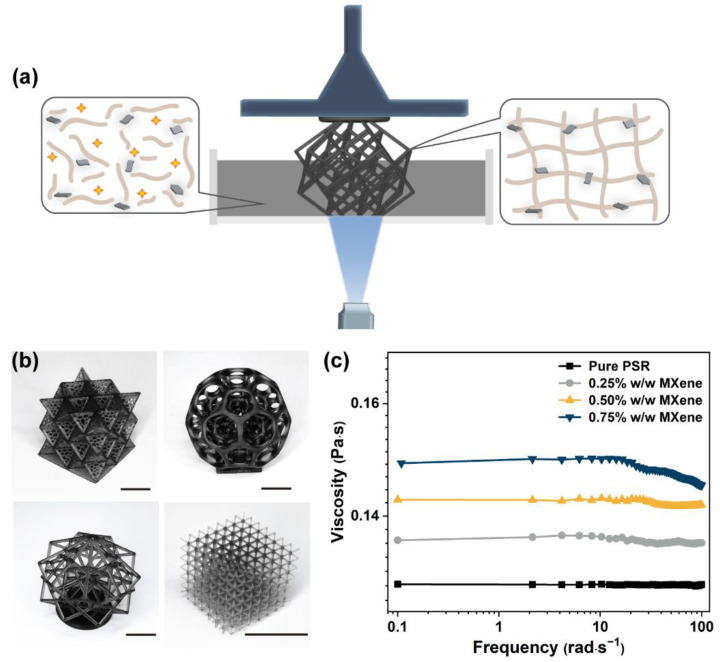
(**a**) Illustration of 3D printing of the MXene nanocomposites using an LCD printer. (**b**) Elaborate structures printed from the PSR/MXene nanocomposites with 0.50% *w*/*w* MXene content (scale bar: 1 mm). (**c**) The viscosity of the liquid PSR and PSR/MXene, each as a function of mutative shear rate.

The exfoliated MXene nanosheets were added to the liquid resin for UV 3D printing; the liquid PAR/MXene could be stable for 36 h at 25 °C without any visible sedimentation (Figure S2). These nanocomposites were fabricated using a commercial LCD 3D printer (Figure 2a). In this LCD-based 3D printing process, the photoinitiator was applied to induce the oligomer and monomer polymerization, after which the MXene nanosheets were shackled in the cured PSR network. Several objects with elaborate structures were manufactured from the PSR/MXene nanocomposites containing 0.50% *w*/*w* MXene (Figure 2b). It should be noted that the rheological properties of the liquid resins are crucial for vat polymerization [25]. Nevertheless, the changes in the viscosity and shear behavior after the absorption of MXene into the PSR are inevitable. As presented in Figure 2c, a slight increase in the viscosity of the PSR/MXene nanocomposites was observed compared to the pure PSR at the same shear rate. Moreover, an increase in the viscosity of the nanocomposites was observed with an increase in the loading of MXene in the nanocomposites. However, the increase in the viscosity was insignificant because of the small amounts of fillers added. Compared to other UV-curable resins for LCD printing [26,27], the viscosity of PSR/MXene used in this study was within the acceptable range. Moreover, no change in the viscosity of pristine PSR was observed for the shear rates ranging from 0.1 to 100 rad·s^−1^, demonstrating the pure PSR to be a Newtonian fluid. At lower values of MXene loading (≤0.5% *w*/*w*), the PSR/MXene nanocomposites showed minimal changes in their viscosities with the shear rate. The rheological characteristics of the slurry, such as thixotropy and shear thinning, were observed at relatively higher shear rates and 0.75% *w*/*w* MXene, suggesting that incorporating large amounts of MXene changed the PSR fluid type. The shear-thinning behavior in vat photopolymerization 3D printing was in agreement with the literature [28]. Accordingly, the designed PSR/MXene nanocomposites exhibited potential for current commercial LCD 3D printers.

### 3.3. Mechanical Properties

**Figure 3:**
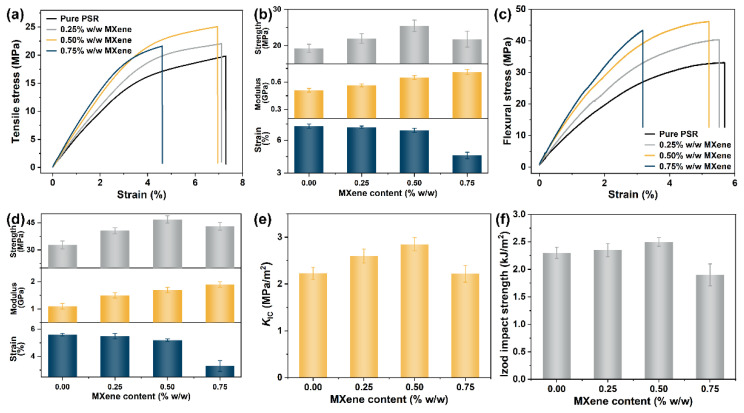
(**a**) The stress–strain curves from the tensile tests. (**b**) Ultimate tensile strength, modulus, and strain at break. (**c**) Stress versus strain from the flexural tests of the pristine PSR and PSR/MXene nanocomposites. (**d**) Ultimate flexural strength, modulus, and strain at break. (**e**) Fracture toughness (*K*_IC_) and (**f**) Izod impact strength for each of the PSR/MXene nanocomposites at various loadings of MXene.

Various industrial applications require diverse 3D printed parts with different mechanical properties: soft to hard or rigid to tough. The tensile behaviors of the 3D-printed samples were investigated, and the actual stress–strain curves from the tensile tests of the pure PSR and PSR/MXene nanocomposites are plotted in Figure 3a. To evaluate the tensile performances of the samples, the ultimate tensile strength, Young’s modulus, and elongation at break were investigated (Figure 3b and Table 1). A certain degree of improvement in the tensile properties of the samples was observed with the incorporation of the MXene nanosheets. The ultimate tensile strength of the nanocomposites initially increased and then deteriorated as the amount of MXene increased. The nanocomposites with 0.5% *w*/*w* MXene exhibited the highest tensile strength (∼32% increase). At 0.75% *w*/*w* MXene, the tensile strength (21.8 MPa) of the nanocomposites decreased, but retained a slightly higher value than that of the pure PSR (19.3 MPa). It was observed that Young’s modulus of each the PSR/MXene nanocomposites was enhanced with increasing MXene concentration, which could be attributed to the high stiffness of MXene compared with that of the polymer matrix. Interestingly, the improvements in the tensile strength and modulus were not associated with a significant reduction in the elongation at break. A slight decline in the elongation at a break of 5.5% was observed in the nanocomposites containing 0.5% *w*/*w* MXene. This may be attributed to the lamellar barrier effect of MXene, which would restrict the polymeric segmental relaxation and cause a decrease in strain to a certain degree [29]. However, due to the breakdown of the hydrogen bond, the interface debonding slightly enhanced the elongation at break [30], leading to slight strain degeneration.

In addition to the tensile behavior, the flexibility of the as-prepared PSR/MXene nanocomposites was explored to further determine the effect of MXene. The stress–strain curves of the flexural behavior of the PSR/MXene nanocomposites are displayed in Figure 3c, and the detailed data, including the ultimate flexural strength, modulus, and strain at break, are listed in Figure 3d and Table 2. Although the tri-functional monomer (SR494 NS) introduced in this study could improve the flexural strength by increasing the crosslinking density in the PSR, the flexural strength (33.0 MPa) of the pristine PSR was extremely low, and impractical for meeting application requirements. Similarly to the tensile behavior, the flexural strength of the PSR/MXene nanocomposites tended to increase and then decrease as the MXene loading increased. As expected, the flexural fracture strength of the nanocomposites containing 0.5% *w*/*w* MXene reached the optimal value of 46.4 MPa, an improvement of 42.7%. With the introduction of MXene, the strain at a break of the nanocomposites gradually decreased as the loading increased (≤0.5% *w*/*w*) and significantly reduced at 0.75% *w*/*w* MXene. The experimental results suggest that an optimal amount of MXene would enhance the strength and modulus (including the tensile and flexural properties) of the PSR while maintaining the strain at break, indicating constant toughness.

To evaluate and confirm the above inference, the toughness values of the pure PSR and PSR/MXene nanocomposites were examined through single-edge notch three-point bending (SENB) tests. The fracture toughness (*K*_IC_) was obtained from the SENB tests (Figure 3e). The *K*_IC_ of the PSR/MXene nanocomposites with 0.5% *w*/*w* was 2.85 MPa/m^2^; compare that with the 2.23 MPa/m^2^ of the pure PSR: a 27.8% increase. The fracture toughness of the nanocomposites decreased to 2.22 MPa/m^2^ at 0.75% *w*/*w* MXene, which was approximately equal to that of the pure PSR, suggesting that incorporating MXene would have no substantial influence on the toughness of the PSR. A similar result could be observed for the Izod impact strength (Figure 3f).

**Figure 4:**
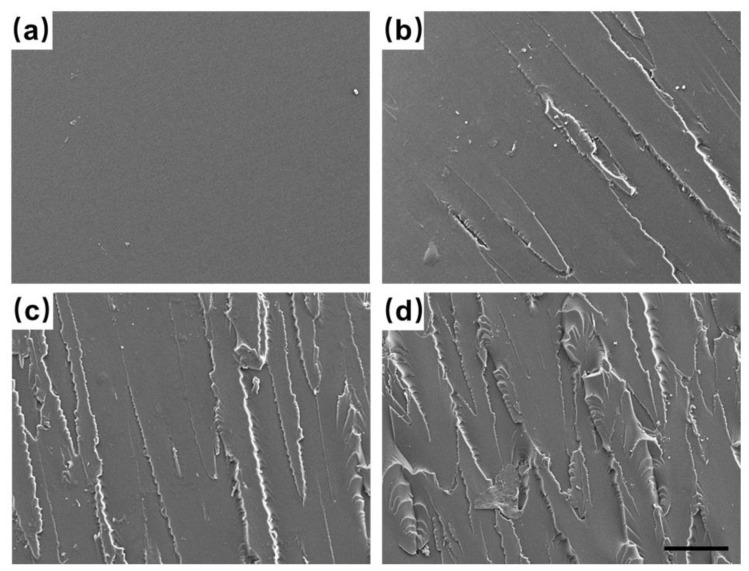
SEM micrographs of the fracture surfaces after the tensile tests of the (**a**) pure PSR; and PSR/MXene nanocomposites with (**b**) 0.25% *w*/*w*, (**c**) 0.5% *w*/*w*, and (**d**) 0.75% *w*/*w* MXene nanosheets (scale bar: 40 μm).

To further explore the mechanistic effect of MXene on the nanocomposites, the fractured surfaces obtained after tension were observed using SEM (Figure 4). The fracture surfaces of the pure PSR were smooth and glassy (Figure 4a), which could have been due to the uniform internal stress distribution in the absence of the nanofillers. During the fracture process, cracks rapidly propagated along the interface vertical to the force applied, leading to relatively low tensile strength (as observed from the tensile tests of Figure 3a). The nanocomposites were comparatively coarser, having rougher fracture surfaces (Figure 4b–d), because of the several linear and nonlinear slits. Furthermore, the fracture surfaces became more uneven and more wrinkled at higher MXene loadings. Owing to the presence of MXene in the nanocomposites, which were considered stress concentrators, the cracks were propagated along an obstructed path in the spatial direction, leading to the formation of several microcracks during the fracture process. Hence, more fracture energy could be absorbed in the nanocomposites, as reflected by the higher tensile strength at the macro level. Moreover, several MXene nanosheets located in front of the cracks (marked with arrows in Figure S3a,c) indicated that the incorporated MXene effectively prevented the growth of the cracks in the PSR. The small holes in the fracture (Figure S3b) indicated that the MXene nanosheets was pulled out from the PSR matrix during the disruption, allowing for energy absorption. However, at 0.75% *w*/*w* MXene, it became difficult to disperse the MXene mass uniformly in the PSR matrix. The agglomeration of the MXene caused the formation of microdefects (Figure S3d), resulting in the deteriorated mechanical properties.

**Figure 5:**
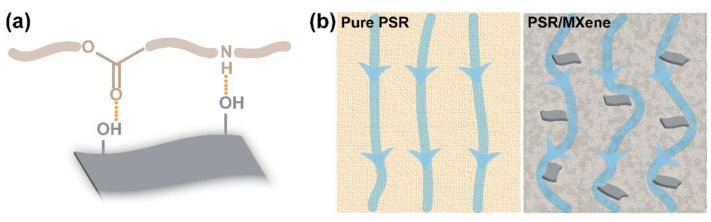
(**a**) Schematic illustrating the interactions between the polymer chain in the PSR and MXene nanosheet. (**b**) The transferred stress path under fracture in the pure PSR and its nanocomposite.

Based on the above analysis, incorporating MXene at an optimal amount of approximately 0.5% *w*/*w* enhanced the mechanical performance of the PSR, which could be attributed to the following two reasons. First, the strong interactions between the PSR and MXene (the correlative schematic is shown in Figure 5a) in the form of hydrogen bonding of the -OH groups of MXene with the oligomers or monomers in the PSR matrix [31]. Moreover, the strong interface interactions may allow the nanocomposites to effectively bear the stress and adequately transfer the destructive energy to the nanocomposites (Figure 5b). Second, the existence of MXene with a two-dimensional scale could restrict the movement of the polymer chains [32,33]. In part, the physical crosslinking in the nanocomposites could enhance mechanical strength. Nevertheless, a higher content of MXene (0.75% *w*/*w*) would lead to its agglomeration in the PSR matrix (Figure S4b). Thus, the mass of the nanofillers affected the photopolymerization of the polymer matrix, resulting in a lower crosslinking density (observed in the DMA tests) and deterioration in the mechanical strength of the nanocomposites.

**Figure 6:**
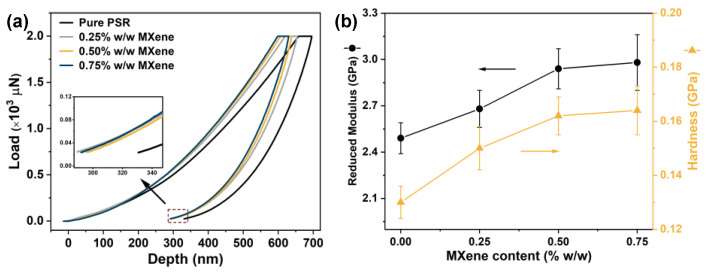
(**a**) Indentation load-depth curves for pristine PSR and its nanocomposites. (**b**) Reduced modulus and hardness of the PSRs with varying MXene content.

In addition to the macro-mechanical properties, micro-mechanical properties are often required. To explore these aspects, nanoindentation analyses were carried out to investigate the mechanical performances of the pure PSR and as-prepared nanocomposites at the macroscale. The load against the depth of the indenter for the PSR/MXene nanocomposites is plotted in Figure 6a, and the data are provided in Table 3. The maximum depths of the nanocomposites were smaller than that of the pure PSR, indicating that the incorporation of MXene effectively improved the micro-mechanical properties. Notably, this ability could have originated from the higher stiffness of the MXene nanosheets than the polymer matrix. However, unlike the overall mechanical properties, which initially increased and then decreased with increasing nanofiller loading, the maximum depths in the nanoindentation tests continued to increase as the MXene content increased (0.75% *w*/*w*); i.e., the trend at the nanoscale test did not fluctuate as that in the macroscale. The reason behind this could be that the micro-mechanical properties of the nanocomposites are not dependent on the flocculation/dispersion of the MXene nanosheets. Moreover, the final depths of the indentation in PSR decreased after absorbing MXene, demonstrating that the nanocomposites have a stronger ability to inhibit plastic deformation. In this context, some additional parameters, such as hardness and reduced modulus, were obtained (Figure 6b). At up to 0.5% *w*/*w* MXene, hardness and reduced modulus values markedly increased, but only slightly increased at 0.75% *w*/*w* MXene (reduced modulus = 2.98 GPa and hardness = 0.16 GPa, corresponding to improvements of 19.7% and 23.1%, respectively). Based on the aforementioned results, the incorporation of MXene would effectively enhance the mechanical performances of pristine PSR.

### 3.4. Thermal Properties

**Figure 7:**
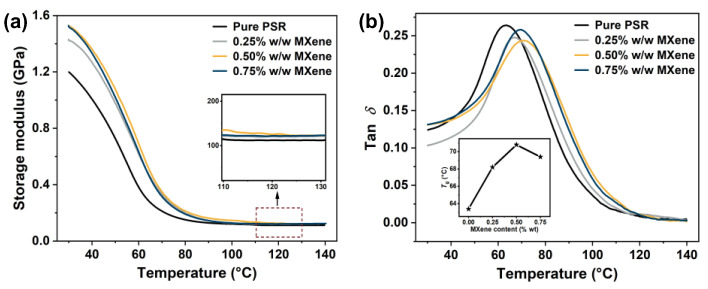
(**a**) Storage modulus and (**b**) tan *δ* for the pure PSR and PSR/MXene nanocomposites. The insets in (**a**,**b**) present the variation in the storage modulus at around 120 °C and *T*_g_ with varying MXene content, respectively.

In addition to the comprehensive mechanical properties, the thermal analysis of the printed objects is crucial for industrial applications. The thermal properties of the PSR and PSR/MXene nanocomposites were determined using DMA. The results are presented in Figure 7 and Table 4. As depicted in Figure 7a, the storage modulus increased from 1199.6 to 1526.5 MPa at 30 °C as the loading of MXene increased from 0 to 0.5% *w*/*w*. However, at 0.75% *w*/*w* MXene, the storage modulus slightly decreased to 1521.0 MPa. Since MXene possessed a higher storage modulus than the pristine PSR, the stiffness of the nanocomposites improved. Meanwhile, arising from the uniform dispersion of a suitable amount of MXene in the PSR (Figure S4a), a reinforced effect due to the nanofillers was achieved. In addition, the strong interactions due to the hydrogen bonding hindered the mobility of the polymer chain around MXene [34], which could have been associated with the higher storage modulus of the nanocomposites. In addition, the crosslinking densities (*υ*_e_) of the pure PSR and PSR/MXene nanocomposites were calculated from the DMA tests [35]. The nanocomposites demonstrated an increase in the crosslinking density compared with the pure PSR (Table 4). However, the crosslinking density of the nanocomposites containing 0.75% *w*/*w* MXene became lower. In the PSR/MXene nanocomposite, the free volumes were occupied by the MXene nanosheets, and the hydrogen bonding compressed the polymer chains, leading to the increased crosslinking density. However, the agglomerated MXene would hinder the polymerization of the PSR, resulting in a lowered crosslinking density [36].

The glass transition temperature (*T*_g_), corresponding to the temperature of the tan *δ* peak, was also obtained from the DMA tests (Figure 7b). The pristine PSR without the nanofillers exhibited a tan *δ* peak at 63.4 °C. By introducing 0.25 and 0.5% *w*/*w* MXene, the *T*_g_ of the nanocomposites increased to 68.2 and 70.8 °C, respectively, which could be attributed to the restricted motion of the polymer chain because of the MXene nanosheets. In addition, the higher degree of hydrogen-bond-based crosslinking between MXene and the polymer chain was beneficial for increasing the *T*_g_ [37]. At 0.75% *w*/*w* MXene, the *T*_g_ value decreased to 69.4 °C, which might have been due to the poor dispersion of MXene at higher loadings.

**Figure 8:**
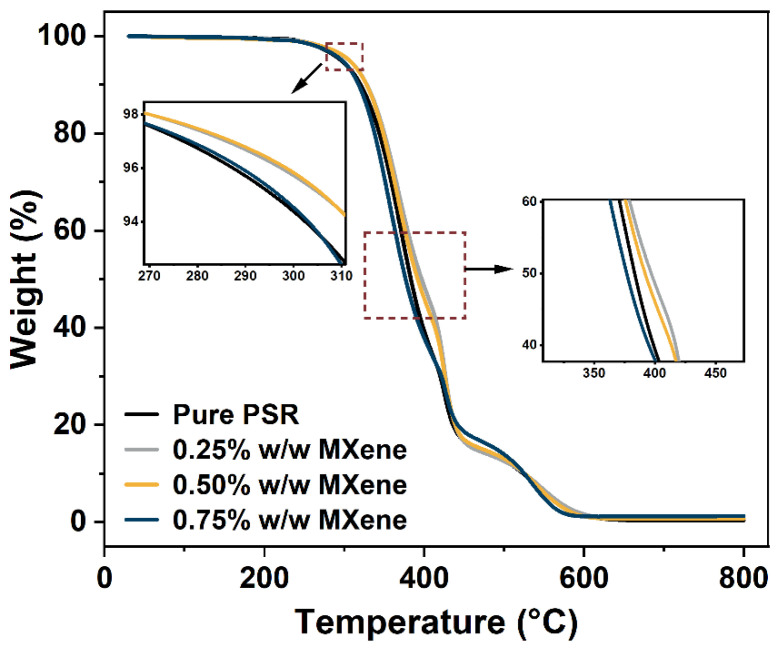
TGA curves of the pristine PSR and PSR/MXene nanocomposites. The insets illustrate the variations around the masses at *T*_−5%_ and *T*_−50%_.

The thermal degradation behavior of the nanocomposites was evaluated using TGA. The thermograms of the PSR and PAR/MXene nanocomposites are depicted in Figure 8. The TGA data, including the temperature at 5% weight loss (*T*_−5%_), the temperature at 50% weight loss (*T*_−50%_), and char residues at 800 °C, are displayed in Table 5. The thermal stability of the PSR and its nanocomposites in terms of weight loss occurred in the range of 200–600 °C. The *T*_−5%_ and *T*_−50%_ of the pristine PSR were 295.6 and 383.2 °C, respectively. By incorporating MXene at relatively lower loadings of 0.25% and 0.5% *w*/*w*, the *T*_−5%_ and *T*_−50%_ values of the nanocomposite were significantly improved. The optimum value of *T*_−5%_ at 0.5% *w*/*w* of MXene was approximately 306.2 °C. In comparison, the maximum *T*_−50%_ was 396.9 °C in the nanocomposite containing 0.25% *w*/*w* MXene, indicating that the motility of the polymer chains might be affected by the introduced MXene nanosheets during thermal degradation. Further, MXene would hinder the diffusion of the volatile components and heat transfer [38]. The strong interactions between MXene and the PSR matrix delayed the scission of the polymer chains, resulting in improved thermal resistance of the PSR/MXene nanocomposites. Comparatively, the *T*_−__5%_ and *T*_−50%_ values decreased at the concentration of 0.75% *w*/*w* MXene. In addition, compared to pure PSR, the char yields of the nanocomposites increased with increasing MXene content. The presence of Ti and C from MXene would have contributed to the char yield. In addition, a large amount of titanium dioxide could be generated at high temperatures [39]. The addition of MXene affected the thermal decomposition of residues of the PSR, leading to the attachment and retention of the char yield, as previously reported [40,41].

## 4. Conclusions

In summary, the MXene-reinforced PSR nanocomposites for LCD 3D printing were successfully prepared. The rheological investigations indicated that the incorporation of MXene slightly increased the viscosity of the liquid resin matrix. The shear thinning behavior was observed with a high loading of MXene at 0.75% *w*/*w*. The PSR/MXene could have great potential for current commercial LCD printers. The incorporation of the MXene nanosheets significantly improved the mechanical and thermal performances of the PSR at the optimum loading of approximately 0.5% *w*/*w*. At even a higher MXene loading (0.75% *w*/*w*), the performance of the PSR deteriorated, which could have been because of the agglomeration of the excess MXene nanosheets. The PSR/MXene nanocomposites could be considered a viable alternative to pure PSRs for a wide variety of applications that warrant high-performing products. In conclusion, we firmly believe that the designed composites would broaden the applications of vat-photopolymerization-based 3D printing.

## Figures and Tables

**Table 1:** Detailed tensile data of the pure PSR and PSR/MXene nanocomposites.

Sample	Tensile Strength (MPa)	Tensile Modulus (MPa)	Elongation at Break (%)	Improved Strength (%)
PSR	19.3 ± 1.1	510 ± 20	7.3 ± 0.2	--
0.25% *w*/*w* MXene	22.0 ± 1.3	565 ± 16	7.2 ± 0.1	14.0
0.50% *w*/*w* MXene	25.5 ± 1.6	650 ± 21	6.9 ± 0.2	32.1
0.75% *w*/*w* MXene	21.8 ± 2.2	712 ± 28	4.6 ± 0.3	12.9

**Table 2:** Detailed data of the pure PSR and PSR/MXene nanocomposites from the flexural tests.

Sample	Flexural Strength (MPa)	Flexural Modulus (GPa)	Strain at Break (%)	Improved Strength (%)
PSR	32.8 ± 2.2	1.1 ± 0.1	5.6 ± 0.1	--
0.25% *w*/*w* MXene	40.6 ± 1.7	1.5 ± 0.1	5.5 ± 0.2	23.8
0.50% *w*/*w* MXene	46.8 ± 2.0	1.7 ± 0.1	5.2 ± 0.1	42.7
0.75% *w*/*w* MXene	43.1 ± 2.1	1.9 ± 0.1	3.3 ± 0.4	31.4

**Table 3:** Detailed data of the pure PSR and PSR/MXene nanocomposites from nanoindentation analyses.

Sample	Max Depth (nm)	Final Depth (nm)	Hardness (GPa)	Reduced Modulus (GPa)
PSR	659.3 ± 23.52	330.1 ± 17.51	0.13 ± 0.01	2.49 ± 0.1
0.25% *w*/*w* MXene	619.0 ± 25.53	259.5 ± 17.10	0.15 ± 0.01	2.68 ± 0.12
0.50% *w*/*w* MXene	605.8 ± 24.21	293.0 ± 18.23	0.16 ± 0.01	2.94 ± 0.13
0.75% *w*/*w* MXene	598.9 ± 22.40	289.8 ± 19.17	0.16 ± 0.01	2.98 ± 0.18

**Table 4:** The detailed data of the pristine PSR and PSR/MXene nanocomposites from the DMA study.

Sample	Storage Modulus (MPa)		Tan δ Peak Height	*T*_g_ (°C)	*υ*_e_ (mol/m^3^)
	30 (°C)	140 (°C)			
PSR	1199.6 ± 5.4	111.9 ± 1.4	0.264 ± 0.012	63.4 ± 1.2	12,622.6
0.25% *w*/*w* MXene	1424.1 ± 7.2	123.9 ± 1.0	0.247 ± 0.015	68.2 ± 0.9	12,774.7
0.50% *w*/*w* MXene	1526.5 ± 6.3	124.0 ± 0.9	0.243 ± 0.010	70.8 ± 1.0	14,071.2
0.75% *w*/*w* MXene	1521.0 ± 3.1	124.2 ± 1.0	0.258 ± 0.012	69.4 ± 0.8	12,958.9

**Table 5:** Detailed data of the pure PSR and PSR/MXene nanocomposites from TG.

Sample	*T*_−5%_ (°C)	*T*_−50%_ (°C)	Residue (%)
PSR	295.6	383.2	0.38
0.25% *w*/*w* MXene	305.7	396.9	0.74
0.50% *w*/*w* MXene	306.2	391.8	0.77
0.75% *w*/*w* MXene	297.0	376.6	1.26

## Data Availability

The data presented in this study are available in this article.

## Supplementary Materials

The following supporting information can be downloaded at: https://www.mdpi.com/article/10.3390/nano12162862/s1. Figure S1: High-resolution TEM image of the exfoliated MXene nanosheets. Figure S2: The storing stability of liquid PSR/MXene at 25 °C. Figure S3: SEM micrographs of tensile fracture surfaces of the PSR/MXene nanocomposites. Figure S4: TEM images of ultrathin nanocomposites.
