# Supplementary material for: 3D Printing of Ultrathin MXene toward Tough and Thermally Resistant Nanocomposites
Source: Nanomaterials (Basel). 2022 Aug 19;12(16):2862. doi: 10.3390/nano12162862 (PMC9414167; doi:10.3390/nano12162862)
Supplement: Supplementary file 1 [file nanomaterials-12-02862-s001.zip › nanomaterials-1845775-supplementary.pdf]

nanomaterials

Supplementary Materials

3D Printing of Ultrathin MXene toward Tough and Thermally Resistant Nanocomposites

Yuewei Li [1,2,*], Ranjith Kumar Kankala [1,2], Ai-Zheng Chen [1,2] and Shi-Bin Wang [1,2,*]

1 Institute of Biomaterials and Tissue Engineering, Huaqiao University, Xiamen 361021, China
2 Fujian Provincial Key Laboratory of Biochemical Technology, Huaqiao University, Xiamen 361021, China
* Correspondence: ywli@hqu.edu.cn (Y.L.), sbwang@hqu.edu.cn (S.-B.W.); Tel.: +86-592-616-2326 (S.-B.W.)

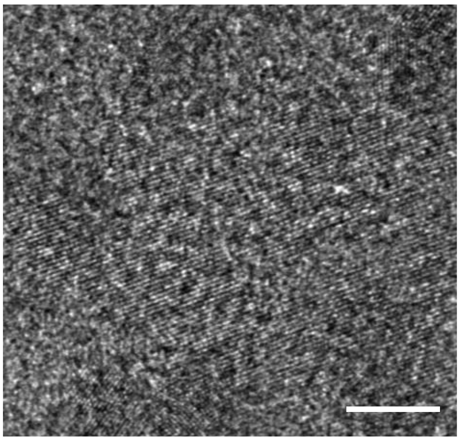

Figure S1. High-resolution TEM image of the exfoliated MXene nanosheets (scale bar: 5 nm).

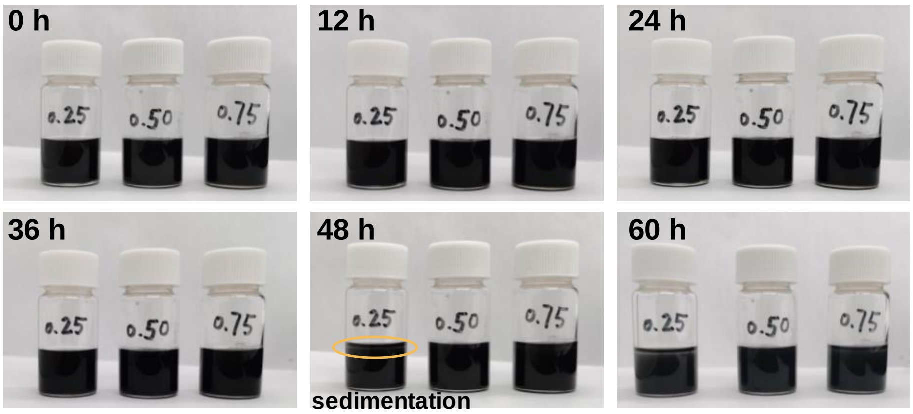

Figure S2. The storing stability of liquid PSR/MXene at 25 °C.

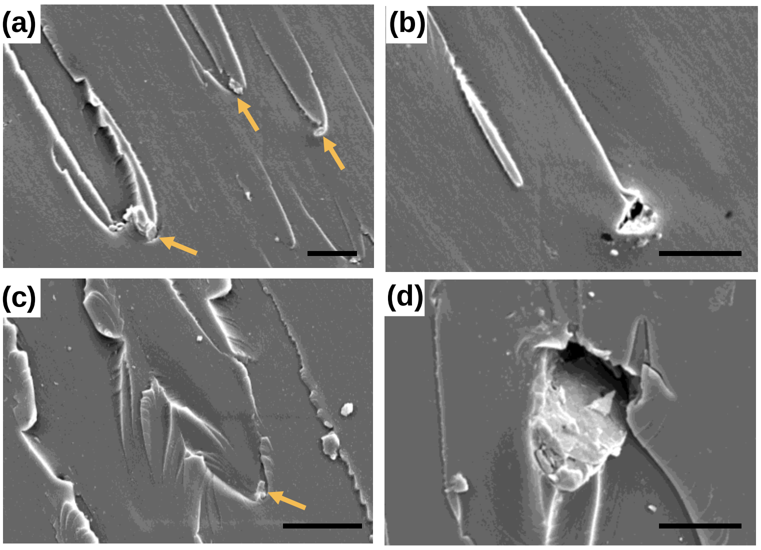

Figure S3. SEM micrographs of tensile fracture surfaces of the PSR/MXene nanocomposites containing (**a**,**b**) 0.5% w/w and (**c**,**d**) 0.75% w/w MXene nanosheets (scale bar: 10 μm).

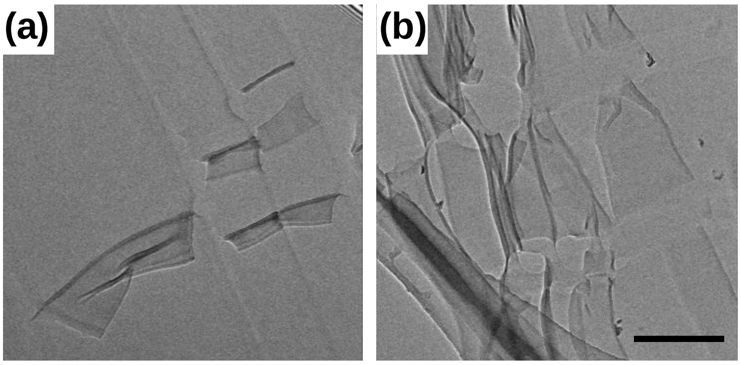

Figure S4. TEM images of ultrathin nanocomposites containing (**a**) 0.5% w/w and (**b**) 0.75% w/w MXene nanosheets (scale bar: 1 μm).
